# PML Differentially Regulates Growth and Invasion in Brain Cancer

**DOI:** 10.3390/ijms22126289

**Published:** 2021-06-11

**Authors:** Maria Tampakaki, Mariam-Eleni Oraiopoulou, Eleftheria Tzamali, Giorgos Tzedakis, Takis Makatounakis, Giannis Zacharakis, Joseph Papamatheakis, Vangelis Sakkalis

**Affiliations:** 1 Institute of Computer Science, Foundation for Research and Technology-Hellas, 70013 Heraklion, Greece; mairata@ics.forth.gr (M.T.); marilena@ics.forth.gr (M.-E.O.); tzamali@ics.forth.gr (E.T.); gtzedaki@ics.forth.gr (G.T.); 2 School of Medicine, University of Crete, 71003 Heraklion, Greece; 3 Institute of Electronic Structure and Laser, Foundation for Research and Technology-Hellas, 70013 Heraklion, Greece; 4 Institute of Molecular Biology and Biotechnology, Foundation for Research and Technology-Hellas, 70013 Heraklion, Greece; makatou@imbb.forth.gr; 5 Department of Biology, University of Crete, 70013 Heraklion, Greece

**Keywords:** Glioblastoma, brain, cancer, PML, in vitro imaging, in silico modeling

## Abstract

Glioblastoma is the most malignant brain tumor among adults. Despite multimodality treatment, it remains incurable, mainly because of its extensive heterogeneity and infiltration in the brain parenchyma. Recent evidence indicates dysregulation of the expression of the Promyelocytic Leukemia Protein (PML) in primary Glioblastoma samples. PML is implicated in various ways in cancer biology. In the brain, PML participates in the physiological migration of the neural progenitor cells, which have been hypothesized to serve as the cell of origin of Glioblastoma. The role of PML in Glioblastoma progression has recently gained attention due to its controversial effects in overall Glioblastoma evolution. In this work, we studied the role of PML in Glioblastoma pathophysiology using the U87MG cell line. We genetically modified the cells to conditionally overexpress the PML isoform IV and we focused on its dual role in tumor growth and invasive capacity. Furthermore, we targeted a PML action mediator, the Enhancer of Zeste Homolog 2 (EZH2), via the inhibitory drug DZNeP. We present a combined in vitro–in silico approach, that utilizes both 2D and 3D cultures and cancer-predictive computational algorithms, in order to differentiate and interpret the observed biological results. Our overall findings indicate that PML regulates growth and invasion through distinct cellular mechanisms. In particular, PML overexpression suppresses cell proliferation, while it maintains the invasive capacity of the U87MG Glioblastoma cells and, upon inhibition of the PML-EZH2 pathway, the invasion is drastically eliminated. Our in silico simulations suggest that the underlying mechanism of PML-driven Glioblastoma physiology regulates invasion by differential modulation of the cell-to-cell adhesive and diffusive capacity of the cells. Elucidating further the role of PML in Glioblastoma biology could set PML as a potential molecular biomarker of the tumor progression and its mediated pathway as a therapeutic target, aiming at inhibiting cell growth and potentially clonal evolution regarding their proliferative and/or invasive phenotype within the heterogeneous tumor mass.

## 1. Introduction

Glioblastoma (GB) is the most malignant and aggressive primary brain tumor, classified as grade IV according to the World Health Organization (WHO; [1]). It is associated with a median survival of 12–15 months [2] and it can appear without a previous tumor diagnosis (primary) or through progression from lower grade tumors (secondary) [3]. Current treatment for GB includes maximal safe resection of the tumor along with adjuvant chemotherapy. However, despite multimodality treatment, GB remains incurable, due to its complex biology, its extensive inter- and intra-tumor heterogeneity, as well as its intra-axial infiltration. In the latter case, GB cells infiltrating brain parenchyma often escape surgical resection and cause recurrence of the disease. Therefore, identifying biomarkers implicated in the physiology of GB are crucial for better understanding its biology and for discovering new therapeutic targets that along with the existing ones may enhance treatment efficacy.

Recent evidence set the Promyelocytic Leukemia Protein (PML) as a crucial regulator of GB evolution that could potentially act as a promising biomarker. PML was originally identified in Acute Promyelocytic Leukemia (APL), as a chromosomal translocation between the chromosomes 15 and 17 [4]. The PML gene is organized into nine exons and, with alternative splicing, it can produce seven isoforms of the protein [5], which vary from PML I (longest) to PML VII (shortest). PML is also an essential organizer of the sub-nuclear structures of the interchromosomal matrix called “nuclear bodies” (NBs) [6]. NBs act as a regulatory hub via interaction with transcription factors and chromatin regulators, thus regulating many functions of the cells such as DNA-damage repair, angiogenesis, viral protection etc.

Apart from APL, modifications in PML expression have been associated with various types of malignancies. However, the specific role of PML in cancer remains to be explained, as PML can have both tumor-promoting or tumor-inhibiting effects regarding the type or the tissue origin of the tumor [7,8].

More specifically, in the brain, PML has been shown to participate in the forebrain development [9,10]. It has been found that PML expression is limited to the neural progenitor/stem cells (NPCs) in the developing mouse cortex [9] and it regulates the NPC physiological migration from the sub-ventricular zone to the olfactory bulb through the rostral migratory stream [11]. This NPC-migration is achieved via a PML-mediated suppression of Slit proteins [11] which are fundamental components in brain circuits development and axon guidance [12]. PML controls the PML/Slit axis via the Polycomb repressive complex 2 (PRC2) [11] and more specifically, its catalytic subunit Enhancer of Zeste Homolog 2 (EZH2), responsible for H3K27 trimethylation resulting in the formation of heterochromatin [13].

Neurogenesis and GB expansion follow common migratory routes [14,15] and there is evidence that these two processes also share common cellular pathways such as the PML/Slit migratory axis [11]. Interestingly, the migrating NPCs are also hypothesized to serve as the cell of origin for malignant gliomas in the adult mouse brain [16], indicating a potential role of PML in GB pathophysiology. In line with these observations, PML expression is often reduced in diverse tumor types including brain tumors, and this is usually connected with aggressive tumor behavior [17]. However, some cases showed an inverse correlation between PML levels and glioma proliferation markers [18]. In particular, contradicting evidence showed growth inhibition of glioma cells by PM I overexpression [18] or an increase in tumor cell growth by PML loss in murine NPC-derived gliomas [19]. Strikingly, PML loss was also found to have a growth promoting effect in normal NPCs [11]. These studies exemplify the complex and often opposing effects of PML in normal- or tumor- cell biology [19].

Mathematical mechanistic models are commonly used to allow deeper understanding and quantification of the underlying biological mechanisms involved in tumor progression [20] and even proposals for clinical translation [21]. A variety of mathematical models have been proposed to describe tumor spheroids and explain their growth dynamics [22,23,24,25]. In terms of their mathematical basis, these models usually describe the tumor volume using discrete or continuous variables. The discrete methods describe cells as individual entities (discrete components) that follow a set of biologically-or experimentally inspired rules, which allow them to interact locally with their environment. In this case, space and time are of discrete nature. Alternatively, the continuum methods approximate tumor cells and their microenvironment as continuous variables commonly described by partial differential equations. It has been proven that the mathematical models can provide invaluable insight of the underlying processes when restricted to experimental observation [22,26,27]. Most existing experimental/computational works focus on either spheroid growth or invasion. Furthermore, these works do not usually consider molecular mechanisms that affect both tumor growth and invasion. It is expected that having a variety of experimental conditions and observables allows for more systemic and robust mathematical model development and parameterization.

In this work, we genetically modified the U87MG GB cell line to conditionally overexpress the PML IV protein. PML IV is the most well-studied isoform and it has been previously identified as a strong growth suppressor in various systems [28,29,30]. However, the role of PML IV in the pathophysiology of GB regarding both the proliferative and infiltrative nature of the disease is still not clear. To further dissect the PML pathway and decipher whether and to what extent the EΖH2 pathway is involved, an EZH2 inhibitor is also used. We study the PML-mediated effects regarding the tumor growth and invasive properties using 2D and 3D biological models and wide field/confocal microscopy. Moreover, a cancer predictive computational model was developed accounting for phenomena such as cellular proliferation, death, motility and phenotypic heterogeneity. The model was parametrized in order to best match predictions with observations in all experimental setups and interventions. The mechanistic model thus allows both quantification and interpretation of the biological observations. Our findings indicate that PML OE inhibits growth, while it positively regulates invasion in the U87MG GB cells. The in silico modeling further suggests a mechanism potentially underlying the PML-mediated regulation of invasion.

## 2. Results

We studied the physiology of our own-established doxycycline inducible PML-IV U87MG cell line. The cell growth was monitored both in 2D and 3D, while the invasive properties were studied in 3D, where cell migration was extracellular matrix (ECM)-dependent. Further, to study the PML-driven glioma growth and invasive properties, we employed DZNeP, an EZH2 methyl-transferase inhibitor [29,30]. The non-induced and the doxycycline induced PML-IV overexpressing (PML OE) U87MG cells were treated with DZNeP both in 2D and 3D conditions at a drug dose range of 0.05–40 μM. All experimental results shown here represent the 30 μM concentration, at which the MTT assay indicated that approximately 80% growth inhibition is reached. A single cell-based mathematical model was utilized to describe and understand the spatiotemporal evolution of the tumor spheroids in all experimental conditions.

### 2.1. Proliferation/Doubling Time Estimation

In principle, glioma cells proliferate with a doubling time ranging from 24 h to several days [31]. The proliferation time of the non-induced U87MG cell line was approximately estimated at 75.2 h ± 12.8 h (mean and SEM) and the intrinsic cell death rate 24% ± 1.5% (mean and SEM). The U87MG-PML OE cells exhibited slower growth dynamics with a doubling time at about 112.2 h ± 17.8 h (mean and SEM) and cell death rate remained at 24.7% ± 1.9% (mean and SEM). Cell-cycle analysis by flow cytometry showed a prolonged S-phase and reduced G2-phase in the U87MG-PML OE cells (Supplementary Figure S1).

### 2.2. Tumor Growth Expansion

In order to mimic the growth of an avascular in vivo GB tumor, 3D multicellular spheroids were generated using the hanging-drop technique [32]. Optical microscopy was used to monitor the growth of the spheroids over time. Bright-field images were captured in predefined time intervals, in order to estimate the area of expansion of the tumor. In order to better assess the physiology of the growing 3D tumors, we used confocal microscopy scans to visualize the cell death pattern and the PML distribution.

**Figure 1:**
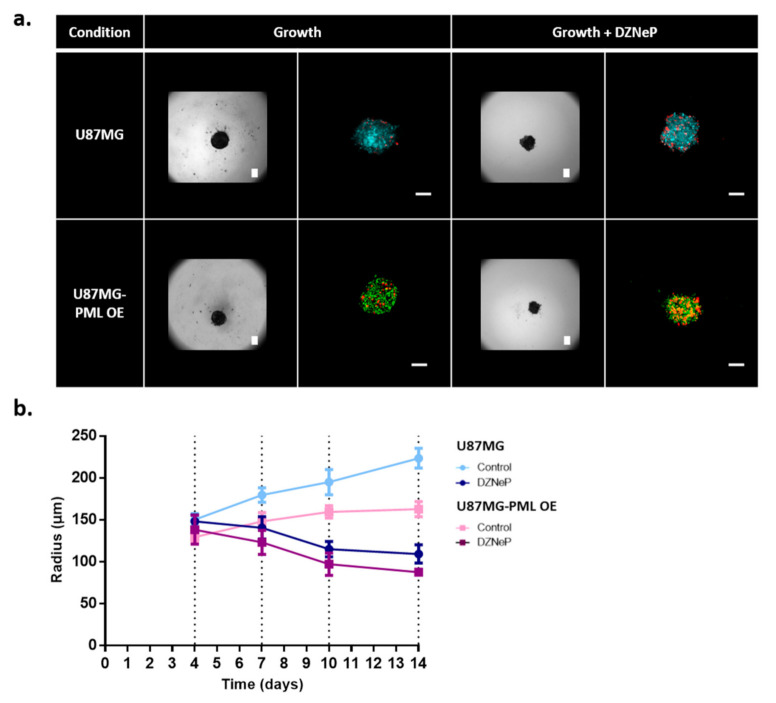
Growth evolution of the non-induced U87MG and the PML-OE (U87MG-PML OE) spheroids. (**a**) Representative bright-field images and confocal z-stacks maximum intensity projection scans of the spheroids in the hanging-drop with and without 30 μM DZNeP treatment (growth; growth + DZNeP, respectively). All images depict the spheroids 3 days after their formation and DZNeP treatment. Green represents DsRed-PML IV, red represents DRAQ7 and cyan represents H_2_DC-FDA. Scale bar is set at 100 microns. (**b**) Spatiotemporal growth curves representing the radial expansion of the spheroids in the hanging-drop overtime in control and drug treatment condition with DZNeP at 30 μM concentration.

The non-induced and the PML OE-U87MG cells approximately needed 4 days from plating (zero day) to spheroid formation. U87MG-PML OE cells exhibited slower aggregation capacity, with deformed tumor boundaries and less compactness as depicted in Figure 1a and in Supplementary Figure S2.

As previously reported [33,34], the presence of an ECM-like substrate in low concentration within the growth environment of the spheroid enabled better cell adhesion and aggregation. Therefore, in order to facilitate the spheroid generation for comparison purposes, we added 1% of BME Pathclear (Amsbio, Cultrex^®^, Abingdon, UK) within the hanging drop, 2 days after seeding. As can be seen in Figure 1a, the necrotic cells are randomly distributed within the tumor mass in all conditions.

The spheroids were monitored for 10 days after their formation. The growth curves in Figure 1b represent the radial expansion over time, as estimated by segmenting the bright-field images. The U87MG-PML OE cells exhibited significantly slower growth (two-sample *t*-test; *p*-value < 0.0001 across all time points) as also indicated in the 2D experiments. EZH2 inhibition alone or in combination with PML OE caused spheroid evolution to similar levels indicating that PML does not further potentiate the DZNeP toxicity (Figure 1b).

### 2.3. Tumor Invasion

To monitor the invasive properties of the non-induced and the PML OE- U87MG cells, the invasion assays were employed by transferring the spheroids in an ECM-like substrate environment within a U-bottom plate on the day of spheroid formation (day 4). Optical microscopy was used every 24 h to monitor the invasive pattern of the spheroids of each cell type up to 96 h of invasion. The invasive capacity of the spheroids was estimated by both the morphological characterization of the pattern adopted and the extent of the invasive rim radius.

**Figure 2:**
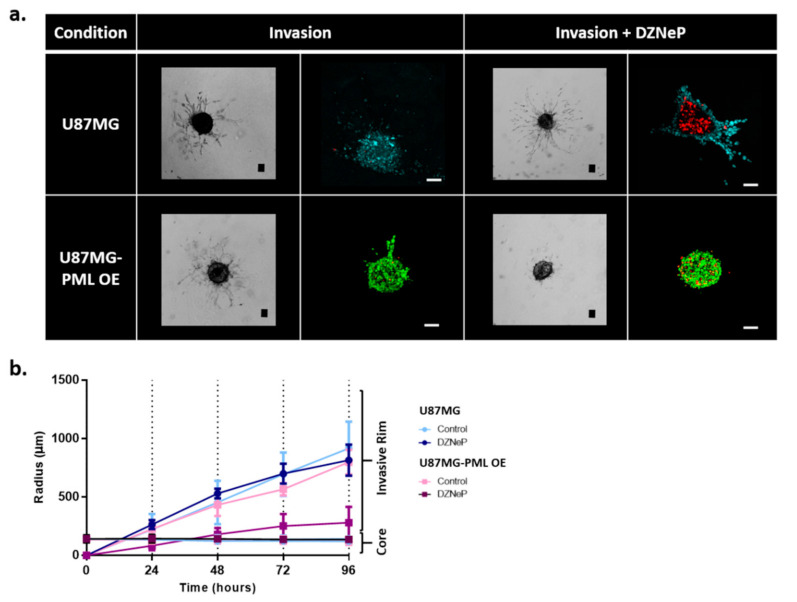
Invasion of the non-induced U87MG and the PML-OE (U87MG-PML OE) spheroids. (**a**) Representative bright-field images and confocal z-stacks maximum intensity projection scans of the spheroids in the invasive ECM-depended condition with and without 30 μΜ DZNeP treatment (Invasion; Invasion + DZNeP, respectively) 72 h after spheroid formation and DZNeP treatment. Green represents DsRed-PML IV, red represents DRAQ7 and cyan represents H_2_DC-FDA. Scale bar is set at 100 microns. (**b**) Spatiotemporal evolution curves representing the radial expansion of the core and the invasive rim of the spheroids within the ECM-depended condition overtime in control and drug treatment condition with DZNeP at 30 μM concentration.

In Figure 2a, the invasive morphology of the non-induced and the PML OE-U87MG spheroids is shown. Both bright-field and confocal images of representative spheroids 72 h after the initiation of invasion are illustrated in Figure 2a with the radial expansion of the spheroid core and the invasive rim over time until the last point of experiment are shown in Figure 2b.

We observed that both the non-induced and the PML OE cells exhibited the typical starburst invasive morphology, commonly followed by the U87MG cells [23]. We also observed that although the growth dynamics of the U87MG-PML OE cells were slower, they exhibited similar invasive rim radial expansion over time as depicted in Figure 2b. In addition, the bright-field images and the confocal scans in Figure 2a qualitatively indicate similar invasive rim density in both conditions. Interestingly, we observed that upon EZH2 inhibition the U87MG-PML OE cells completely lost their ability to migrate, while DZNeP or PML –OE alone cells remained almost unaffected. Note, in Figure 2b that the invasive rim of the DZNeP-treated U87MG-PML OE spheroids is at core level, while the non-induced U87MG treated spheroids did not differ from the control, untreated ones, as also indicated in the confocal scans in Figure 2a. Significance of differences in the radial expansion of the invasive rim in the U87MG non-induced and U87MG-PML OE spheroids was estimated across all time points in the control and the DZNeP treated conditions, using one-way ANOVA and Tukey-Kramer post-hoc test (*p*-value < 0.05 for 24 h and *p*-value < 0.001 for 48–96 h).

### 2.4. Mathematical Modeling and Analysis of Spheroid Growth and Invasion

A single cell-based mathematical model was developed to describe the evolution of tumor spheroids in different conditions and discriminate the differential behavior between non-induced and PML OE-U87MG cells. The model accounted for phenomena such as cellular proliferation, death, motility and phenotypic ratio among motile and adhesive cells. The model was evaluated based on its ability to accurately simulate the in vitro dynamics of the spheroid core in the growth conditions and the in vitro dynamics of both the core and the invasive rim in the invasive conditions constrained by the experimental data. In order to properly parametrize our mathematical model, a parameter study that investigated the extent at which each parameter affects tumor evolution was conducted. The physiology of the non-invasive and invasive spheroids was investigated, and the mathematical model was built and constrained sequentially in a stepwise manner.

#### 2.4.1. Spheroid Growth Study

At first, in the non-invasive condition, the parameters explored included the proliferation time, the intrinsic/random cell death, the thickness of the proliferating rim (proliferation depth), and the initial cell density. The parameter study showed that decreasing the proliferation time or increasing the proliferation depth resulted in an increase in the tumor population and tumor expansion, as expected (Supplementary Figure S3a,b). Furthermore, increasing the random death reduced the tumor growth, while higher initial cell density increased the tumor growth (Supplementary Figure S3c,d). In the mathematical model, the proliferation time and the intrinsic cell death rate were constrained to be close to their (2D in vitro) experimental values. The proliferation depth and the initial cell density could not be easily derived from the experiments and therefore were chosen in a way that minimized the discrepancy in spheroid expansion between simulations and experiments.

**Figure 3:**
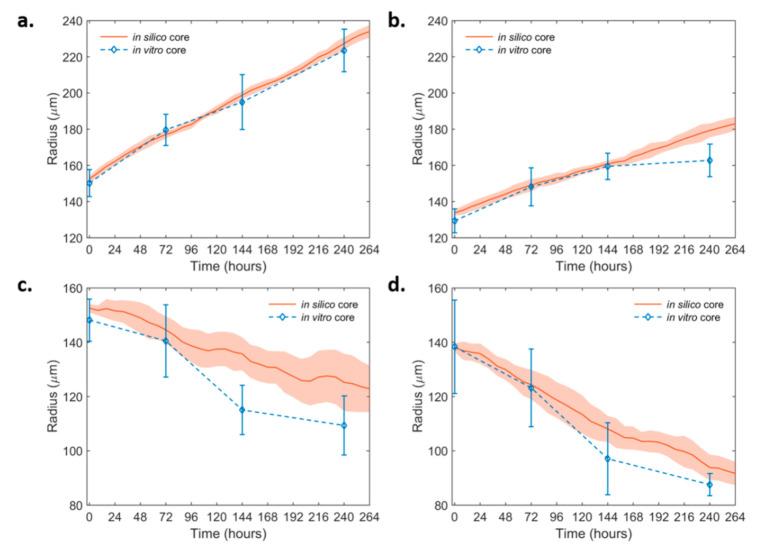
In silico simulation curves at the best-fit values of the growth dynamics of the non-invasive non-induced and the PML OE-U87MG spheroids over time. (**a**) non-induced U87MG and (**b**) U87MG-PML OE (**c**) non-induced U87MG spheroids under 30 μM DZNeP treatment and (**d**) U87MG-PML OE spheroids under 30 μM DZNeP treatment. The in vitro estimates are also shown for comparison.

Based on the biological observations and the thorough parameter study of the modeling parameters that was performed, the parameters that best fit the growth dynamics of the non-induced U87MG in vitro spheroids in the non-invasive condition were selected. Keeping all the rest parameters fixed, variation only in the proliferation time was allowed to describe the altered growth dynamics observed in the U87MG-PML OE cells. The simulations showed that a 60% increase in the proliferation time in the U87MG-PML OE cells relative to the non-induced U87MG cells could well-explain the growth curve of the U87MG-PML OE spheroids, which agreed with the doubling time experiments. We then investigated the (30 μM) DZNeP-mediated death on the growth dynamics. We concluded that a death rate equal to 0.0065 h^−1^, which corresponded to 65% of the proliferation rate of the U87MG cells, could well-explain the growth inhibition observed in both the non-induced U87MG and the U87MG-PML OE spheroids under (30 μM) DZNeP treatment. Note that, for the case of the U87MG-PML OE spheroids, this death rate was approximately equal to their proliferation rate, resulting in their significant growth inhibition and increased overall death. Figure 3 shows the growth curves of the non-induced (Figure 3a,c) and the PML OE- U87MG (Figure 3b,d) spheroids without and with treatment with DZNeP as derived from the experiments and predicted in silico. All the fitted parameters used in the model are summarized in Table 1.

#### 2.4.2. Spheroid Invasion Study 

In order to mathematically describe the invasive capability of both the non-induced and PML OE- U87MG spheroids, it was assumed that cells move randomly (diffusive movement), biased only by their adhesive trait. Specifically, the tumor was composed of cells with different phenotypic traits with respect to their adhesion properties. In particular, we considered phenotypes with very high cell-to-cell adhesiveness (*Adhesive*) and very low adhesiveness (*Motile*). Thus, the invasive pattern exhibited in the simulations was attributed in probabilistic cell movement of different subpopulations regarding their cell-to-cell adhesion properties.

The parameters explored in the invasive condition were the diffusion coefficient of the cells and the phenotypic ratio (Supplementary Figure S4c,d), while the rest were kept constant at their best-fit values estimated previously in the growth condition. Note however that in general, the proliferation and death rates also affected the invasive dynamics and particularly the core dynamics and the number of invasive cells (Supplementary Figure S4a,b). Because diffusion is a random process and the *in silico* tumor is composed of few tumor cells, all the simulations have been repeated 10 times. The mean and standard deviation of the core and invasive rim estimates have been derived from all the experiments performed. It was observed that the diffusion coefficient did not affect the core dynamics. Yet, it significantly affected the dynamics of the invasive rim in a positive way (Supplementary Figure S4c). As the diffusion coefficient increased, the expansion of the invasive rim was higher. We also observed that increasing the ratio of *Motile* phenotypes relative to *Adhesive*, while keeping the diffusion coefficient the same, a profound decrease in the core dynamics and a significant increase in the invasive rim expansion were observed (Supplementary Figure S4d). Note that the invasive rim was affected mainly due to the low number of the invasive cells. The core dynamics, on the other hand, were drastically reduced by increasing the *Motile:Adhesive* ratio, as more cells are allowed to move away from the tumor core. The *Motile:Adhesive* ratio also regulated the density of invasive cells (Supplementary Figure S5). Note also that the adhesive property of the cells did not affect the growth dynamics of the proliferative conditions; it only affected the expansion (core and invasive rim) dynamics in the invasive conditions.

**Figure 4:**
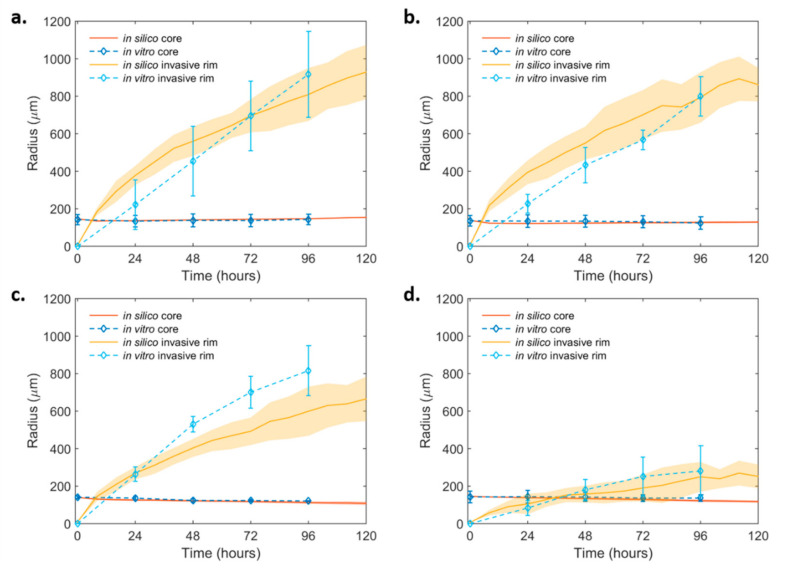
In silico simulation curves at the best-fit values of the invasive dynamics of the non-induced and the PML OE- U87MG spheroids over time. (**a**) Non-induced U87MG and (**b**) U87MG-PML OE; (**c**) non-induced U87MG spheroids under 30 μM DZNeP treatment and (**d**) U87MG-PML OE under 30 μM DZNeP treatment. The in vitro estimates are also shown for comparison.

In order to come in agreement with the in vitro invasive experiments, a diffusion co-efficient equal to 2.00 × 10^−9^ cm^2^/s and an initial ratio of *Motile:Adhesive* approximately equal to 2:1 could well-describe the expansion dynamics of the core and the invasive rim of both the non-induced and the PML OE spheroids (Figure 4a,b). Note that the growth-dependent parameters as derived from the growth condition were kept fixed in the invasive conditions for the non-induced and PML OE spheroids. The parameters involved in the invasive condition are also shown in Table 2.

The presence of DZNeP in the invasive conditions was assumed to act with the same death rate as in the non-invasive conditions. Here, we investigated its potential role on the invasive expansion as reflected in the diffusion coefficient and *Motile:Adhesive* ratio. It was observed that, in the presence of DZNeP, variations in the motility speed alone could not capture both the core and the invasive rim dynamics. Interestingly, we observed that the dynamics of the treated non-induced U87MG cells were well-approximated when the *Motile:Adhesive* ratio changed from 2:1 to 1:1 and the diffusion coefficient remained the same (Figure 4c). On the other hand, we observed that, in order to explain the motility inhibition observed in the U87MG-PML OE cells under DZNeP treatment, a drastic change in the *Motile:Adhesive* ratio was necessary to reach the different dynamics of both the invasive rim and core spheroid, together with a small reduction in their diffusive capacity. In particular, a *Motile:Adhesive* ratio equal to 1:4 and a diffusion coefficient equal to 1.00 × 10^−9^ cm^2^/s best fitted the expansion dynamics, given the constraints of the other parameters from the growth conditions (Figure 4d). Thus, DZNeP eliminated the motile phenotypes in the PML OE condition and reduced the motility of the cells that managed to detach from the tumor core and migrate.

## 3. Discussion

In this work the physiological role of the PML protein in GB cell cultures was investigated. For this purpose, 2D and 3D biological models were applied, imaged with optical and fluorescence microscopy and modeled with mechanistic, single cell-based computational predictive algorithms. In order to better understand and quantify the PML-mediated effect on GB proliferation and invasion. The invasive capacity of the established U87MG glioma cells modified to overexpress the PML protein was investigated. Their physiology in OE and control conditions was observed over time and the specific phenotypes were translated into computational parameters in a custom-made CA individual-cell-based predictive model.

*PML OE inhibits cell aggregation and spheroid formation.* The U87MG-PML OE cells exhibited reduced aggregation capacity. They generated smaller and occasionally deformed spheroids in conventional media, and typically needed a longer time interval for the spheroid formation compared with the controls. We also tested whether the aggregation dynamics of the U87MG-PML OE cells would be affected under varying concentrations of FBS (data not shown). As previously observed, media additives can alter the aggregation capacity in 3D/hanging-drop cell cultures [34]. However, no change in sphere-forming was observed in the U87MG-PML OE cells. The only condition that spheroid formation was facilitated was by the addition of an ECM-like substrate within the hanging-drop, which has been previously suggested to improve cellular aggregation and formation of 3D structures [33,34]. These findings are in line with previous work that reported the inhibitory role of PML IV in cell proliferation and 3D sphere formation of a breast cancer model [28].

*PML OE inhibits tumor growth.* The U87MG-PML OE cells exhibited increased proliferation time compared with the controls in the trypan blue viability assay experiment. Furthermore, as also shown in previous reports [11], PML OE reduced the tumor growth expansion of the 3D spheroids, also indicating lower proliferative capacity. The proliferation time of the cells can be attributed to a variety of factors affecting their growth properties. The spontaneous/intrinsic cell death rate, for example, is a key characteristic of each cell line and contributes to the progression of the tumor. The PML IV isoform is a regulator of apoptosis, senescence and DNA damage by activating the p53 protein [30]. Yet, the trypan blue viability assay, as well as the confocal imaging, revealed no significant differences in the spontaneous cell death rates between the U87MG-PML OE cells and the controls, indicating that the proliferation time prolongation is not attributed to an increased cell death pattern. Flow cytometry analysis for cell-cycle exhibited an increased S-phase fraction, followed by a decreased G2/M-phase, suggesting that the growth inhibition could be attributed to cell-cycle arrest. Our results are in line with previous findings, indicating that PML OE results in decreased proliferation rate and cell-cycle, that used different PML isoforms or biological cancer models [18,28].

*PML maintains the migratory capacity of the tumor cells.* PML OE altered the invasive properties of the U87MG-PML OE cells. The invasive pattern was common in both cell lines adopting the typical starburst morphology [23]. However, in spite of their slower proliferation rate, PML-overexpressing cells presented similar invasive evolution in terms of both expansion and cellularity of the invasive rim.

Even though the core of the U87MG-PML OE spheroids was smaller and deformed over time, the border of the invasive rim was the same compared with the non-induced U87MG spheroids. The U87MG-PML OE tumor core exhibited a slight reduction in size during the first invasive time points. This slight reduction is possibly attributed to both higher migratory and a lower proliferative capacity of the U87MG-PML OE cells and underscores the opposing roles of PML in cell growth and migration. This implies an uncoupling of cell proliferation and migration that would allow non proliferative tumor cells to maintain their metastatic properties, a process that resembles the dormancy state of micro metastatic cells.

*PML mediates tumor growth and invasion through distinct cellular mechanisms*. Amodeo et al. [11] using PML knock-out mice that lack all PML isoforms, proposed that PML regulates tumor migration via a PML/ΕΖH2/Slit axis [11]. To further dissect the EZH2 pathway regarding GB growth and invasion, we used the DZNeP inhibitor of the EZH2 and studied its effects on cell migration and proliferation. Various concentrations of DZNeP showed no growth difference between the U87MG-PML OE and the non-induced cells. This would indicate that the PML induced growth inhibition probably does not depend on the EZH2 pathway. However, regarding the invasive capacity of the cells, the PML OE spheroids completely lost their ability to migrate upon EZH2 inhibition, while the non-induced U87MG spheroids were almost unaffected. The mathematical model quantifies this hypothesis. When the DZNeP sensitivity was assessed in the non-induced U87MG and the U87MG-PML OE invasive spheroids, a significant reduction was shown in the invasive rim of the U87MG-PML OE cells. A possibility is that PML potentiates the effect of DZNeP to depress SLIT expression and inhibition of migration. In other words, this finding indicates that the EZH2-mediated pathway is highly functional for the PML-mediated cell migration regulation. Alternatively, this may also be achieved by another yet unknown biochemical mechanism. Overall, our findings indicate that, in GB, PML inhibits tumor growth while it modulates cellular migration and these two PML-driven functions are mediated by distinct cellular mechanisms. The mathematical simulations support this finding and also propose a potential mechanism to explain the biological observations.

*In silicosimulations indicate a mechanism of action.* Overall, the parameter study verifies the role of specific physiological parameters in tumor expansion and quantifies the in vitro observations. A minimal as possible model was built to simulate the growth and invasion of both the non-induced and the PML-OE spheroids with and without the presence of the EZH2 inhibitor, aiming to understand and quantify the underlying mechanisms involved. The mathematical model, constrained by the parameters derived when simulating the growth conditions, predicted that, upon EZH2 inhibition in the PML OE cells, a reduction in the diffusion coefficient and a significantly large switch of many cells in the adhesive state is required to explain the observations in the invasive condition. More specifically, upon EZH2 inhibition in the PML OE cells, the model suggests that the diffusive capacity of the cells is reduced; thus, they invade less distance of the surrounding environment. Furthermore, the migratory phenotypes are eliminated while they switch to a more adhesive state; therefore, fewer cells tend to leave the initial tumor core and migrate overall. This is also in line with the inhibitory action of EZH2 on E-cadherins, which mediate cell–cell adhesion and regulate the mobility capacity of the cells [35,36].

Similar single cell-based discrete mathematical approaches have been introduced before to describe either the growth [22,31] or the invasion [23,25,37] of multi-cellular tumor spheroids. These approaches have been also compared against in vitro experiments with high accuracy. Usually, in the non-invasive conditions, the spheroid radius was determined at different time points and compared with the in silico predictions under a variety of conditions. Spatial information derived from staining of various components such as cell nuclei, dead cells or/and collagen for ECM has been also utilized to improve the understanding of the underlying processes involved [22]. Similarly, in the invasive conditions both the core radius and the invasive rim were determined over time to describe the expansion of spheroids [23,37]. However, works constrained in both invasive and non-invasive conditions by in vitro experiments are limited [38,39]. Further, none of these works focuses specifically on the effect of PML on the expansion of GB spheroids. In our work, the tumor growth of the non-invasive and the invasive spheroids was investigated, and the mathematical model was built and constrained sequentially in a stepwise manner in order to explain eventually all the experimental observations. The purpose of our work was thus to identify the minimal components that must be included in our mathematical framework to explain the in vitro results.

In our approach, we assumed that cells lie on a regular grid; thus, they were confined to move and proliferate only towards specified directions. We also assumed a homogeneous ECM that allowed cells to migrate evenly to all directions. The potential ECM remodeling mechanisms involved were not taken into account. Furthermore, in our mathematical model, the molecular mechanisms and pathways involved in the overexpression of PML were only phenotypically approached with respect to their observed impact on proliferation, motility and death patterns.

In the future, it would be interesting to explore and incorporate the evolution over time of the concentrations of molecules involved in the PML pathway, thus quantifying the whole pathway and their impact on cellular processes, such as proliferation and motility. Additional local stochastic events, such as stochasticity in the cell-cycle duration and the proliferation depth, can be included to better capture local inhomogeneities in cell behavior. Furthermore, off-lattice approaches, where cells are allowed to move freely in any direction, could be used instead of cellular automata to better approximate cell movement.

## 4. Materials and Methods

### 4.1. Cell Lines

The well-established human GB cell line U87MG (ATCC^®^ HTB-14™) was used in this work. The cells were cultured in Dulbecco’s modified Eagle medium (DMEM; Gibco, Fisher Scientific, Leicestershire, UK) supplemented with Ham’s Nutrient Mixture F12, cytokines (FGF-EGF, Peprotech, London, UK) and B27 (ThermoFisher-Scientific, Waltham, MA, USA), 10% heat-inactivated fetal bovine serum (FBS) and 50 μg/mL gentamycin (PanReac AppliChem GmbH, Darmstadt, Germany; a.k.a. DMEM++). Cell cultures and subsequent experiments were incubated in standard lab conditions (37 °C, 5% CO_2_, 95% humidity).

Regarding the genetic modifications, the cells were infected with an rtTA expressing lentivirus plasmid (pLenti CMV rtTA3 Blast (w756-1) which was a gift from Eric Campeau (Addgene plasmid # 26429; http://n2t.net/addgene:26429; RRID: Addgene_26429)). After blasticidin selection, they were further infected with a lentivirus plasmid carrying the PML isoform IV, C-terminally fused to the DsRed monomer (Clontech, Mountain View, CA, USA) fluorescent protein (cloned between Xbal and BamHI in PMA2780 (pMA2780 was a gift from Mikhail Alexeyev (Addgene plasmid #25438; http://n2t.net/addgene:25438; RRID:Addgene_25438)) [40]). PML expression was controlled by the TET operator and induced upon activation of rtTA by the antibiotic doxycycline. In order to induce the PML expression, doxycycline was added to the culture medium at a final concentration of 1 ng/mL over time. The presence of the PML protein is verified by the DsRed fluorophore illumination under doxycycline presence and the signal is dotted due to the PML-NBs formation within the cell nucleus.

### 4.2. 2D Cell Cultures. Cell Proliferation, Cell Viability and Cytotoxicity Assays

#### 4.2.1. Trypan Blue Viability Assay

In order to determine cell doubling times and spontaneous cell death, a protocol adopted by Oraiopoulou et al., 2017 [31] was used. In brief, a single cell suspension solution of 20,000 cells/mL was seeded per well in a 24-well plate and incubated for 7 days. Every 24 h after seeding, the culture medium of one well per cell type was removed and a single cell suspension was produced using trypsin-EDTA 0.25% (Merk, Sigma-Aldrich, Darmstadt, Germany) solution. Followingly, 0.4% *w*/*v* trypan blue (T6146, Merk, Sigma-Aldrich, Darmstadt, Germany) solution of equal volume was added to the single-cell suspension for dead cells exclusion. The cell concentration for each cell type was measured within a 24 h interval, using a hemocytometer. The average (and standard error of the mean; SEM) growth rate and proliferation time of each cell line was estimated by applying an exponential linear regression model on the daily cell population data. The spontaneous cell death percentage was estimated as the average percentage of the dead cells compared with the whole cell population.

#### 4.2.2. Flow Cytometry

Flow cytometry was performed for cell-cycle estimations. The cells were labeled with propidium iodide (PI) (PI-Sigma, Sigma-Aldrich, St. Louis, MO, USA) and PI excitation was measured and analyzed using BD flow cytometer (FACS, Becton Dickinson, Franklin Lakes, NJ, USA) and FACS Calibur analyzer (BD Biosciences, San Jose, CA, USA), while the cell-cycle analysis was further performed using the ModFit LT software (Verity Software House, Topsham, ME, USA).

#### 4.2.3. MTT Viability Assay

Indirect estimation of relative cell numbers was conducted by the MTT colorimetry assay. In order to assess the sensitivity to DZNeP (3-Deazaneplanocin A HCl) (Selleck Chemicals LLC (Houston, TX, USA)), the MTT viability assay was performed. For each cell type, a single cell suspension solution of 50,000 cells/mL per well was seeded in a 24-well plate. The cells were treated with the methyl-transferase inhibitor 3-Deazaneplanocin A (DZNeP) (Selleck Chemicals LLC (Houston, TX, USA)) at a dose range of 0.05–40 μM, dissolved in DMSO (PanReac Applichem, ITW Reagents, Darmstadt, Germany). DMSO was used as vector to a final dilution of 1:1000. The plate was incubated up to 90–100% confluence. Followingly, the culture medium was removed from all wells and replaced with 200 μL MTT (M5655-1G, Merk, Sigma-Aldrich, Darmstadt, Germany) in a working solution of 1 mg/mL dissolved in PBS. The plate was incubated at 37 °C in darkness in CO_2_ absence for ~3 h until the formation of intracellular purple formazan crystals. The crystals were solubilized in 300 μL of acidified isopropanol and the optical absorbance of the solution was monitored at 595 nm using a microplate spectrophotometer (ASYS HITECH, Biochrom, UK). Repeated measurements at 660 nm followed, for normalization over cell density. The dose-response curves were generated using the formula:

%growth inhibition = ((control-test value) × 100)/control
(1)

The control corresponds to the viable cell population measurements of the untreated wells.

### 4.3. 3D Cell Cultures

#### 4.3.1. Generation of 3D Multicellular Spheroids

3D multicellular spheroids were generated using the hanging-drop technique, as previously described in [23,41]. In brief, a single cell suspension solution of approximate cell density 625 cells/50 μL of DMEM++ was seeded per well in a 96-well hanging drop plate (3D Biomatrix, Ann Arbor, MI, USA). Agarose solution 1% *w*/*v* was added in the reservoirs of the plate to prevent droplet evaporation. DZNeP drug treatment at 30 μM was performed on the day of spheroid formation (day 4). The growth pattern of the spheroids was monitored over time up to 14 days using Leica DFC310 FX inverse wide field optical microscope (Leica, Munich, Germany).

#### 4.3.2. Invasion Assay

In order to study the invasive physiological characteristics of the different cell types, an invasion protocol previously described by [23] was performed. On day 4 after hanging-drop plating, the aggregation process was completed, the spheroids were formed and transferred in a 96-well U-bottom plate within the invasion solution. The invasion solution contained BME Pathclear (Amsbio, Cultrex^®^, Abingdon, UK) diluted in supplemented DMEM++ at a final concentration of 1:1. Once the spheroids were placed within the U-bottom plate, the U-bottom plate was centrifuged for 5 min at 300× *g* rpm at 4 °C in order to place the spheroids at the center of each well, homogeneously distribute the invasive substrate and eliminate any bubbles within it. After 1 h of incubation at 37 °C for solidification of the invasive substrate, culture medium was added per well and replenished when needed. DZNeP drug treatment at 30 μM was performed on the day of U-bottom plating, once the ECM substrate was solidified and the culture medium was added. The expansion and the invasive morphology adopted by the spheroids was monitored over time for a total period of 96 h using a Leica DFC310 FX inverse wide field optical microscope (Leica, Munich, Germany).

#### 4.3.3. Bright-Field Data Analysis

##### Spheroid Image Segmentation

For all spheroids, the tumor core area was semi-automatically segmented from the image background using the Fiji software. For the invasive spheroids, the evolution of the overall invasive area was estimated by measuring the maximum radius that encloses all the invasive cells taken from the core center to the periphery, as described in [41]. To estimate the invasive rim, the radius of the core of the spheroid was subtracted from the radius of the whole invasive area. Drug sensitivity was assessed by measuring the reduced areal expansion of the treated spheroids compared with the untreated control.

##### Statistical Analysis

Statistical analysis was performed in Matlab (R2009b) 7.9 (The MathWorks Inc., Natick, MA, USA).

##### Tumor Growth Analysis

Significance of the growth differences in the U87MG non-induced and the U87MG-PML OE spheroids across all time-points was estimated using two-sample *t*-test.

##### Tumor Invasion Analysis

Significance of differences in the radial expansion of the invasive rim in the U87MG non-induced and U87MG-PML OE spheroids was estimated across all time points in the control and the DZNeP treated conditions using one-way ANOVA and Tukey–Kramer post-hoc test.

#### 4.3.4. Confocal Imaging and Analysis

For confocal scans an LSM 710, AxioObserver (Carl Zeiss, Oberkochen, Germany) confocal microscope was employed at 10× magnification. Samples were transferred in μ-slide 8-well high ibiTreat plates (ibidi, Munich, Germany). A protocol described in [41] was followed. In brief, the non-invasive spheroids were transferred in the μ-slide plate 72 h upon spheroid formation and immobilized in CyGEL (Biostatus, Shepshed, UK). The invasive spheroids were transferred in the μ-slide plate on the day of spheroid formation and cultured in invasion solution as described above. Drug treatment at 30 μM for the invasive condition was performed within the μ-slide plate. The non-invasive spheroids were treated within the hanging-drop, as described previously, and transferred in the μ-slide plate on the day of scanning. PML bodies were detected as DsRed fusion signals fluorophore, dead nuclei were detected using the DRAQ7 (Biostatus, Shepshed, UK) nuclear probe at 1:200 solution overnight and H_2_DC-FDA (Invitrogen, ThermoFisher-Scientific, Waltham, MA, USA) was added at 5 μM approximately 10 min in the dark before scan, as a counterstain for the cell bodies. Z-stack and maximum intensity projection images were acquired at 543/643/488 nm and analyzed in Zeiss Zen 3.3 (blue edition) software (ZEN Digital Imaging for Light Microscopy, RRID:SCR_013672).

### 4.4. In Silico Modeling

A single cell-based discrete mathematical model was used to simulate spatiotemporally the growth expansion of tumor spheroids implemented in Matlab (R2009b) 7.9 (The MathWorks Inc., Natick, MA, USA). Single, cell-based models have been widely used to describe tumor spheroids and explore several aspects of tumor growth including the evolution of tumor morphology, the impact of phenotypic diversity, tumor-induced angiogenesis, as well as tumor invasion [25]. These models accounted for cell-level interactions that may considerably affect the population dynamics. In the context of this model, each individual cancer cell was described by a discrete cellular automaton (CA) and obeyed in certain rules regarding its proliferation, death and motility [23,31]. The cancer cells were assumed to lie on a regular 2D lattice that resembles a planar slice through the 3D multicellular spheroid. The lattice size *L* equals to 5 mm and each *h x h* lattice site can accommodate only one cell, with size *h* equal to 21.5 μm. Cancer cells were restricted to move and proliferate to nearing sites defined by the lattice. Cells could be found in one of three states: proliferative, quiescent or dead. Cells might proliferate according to the corresponding doubling time and if an adjacent empty neighborhood site was found. Cells that were ready to proliferate but for which there was no space for their daughter cell were stated as quiescent. These cells stayed in that state until an empty space was found. Cells could also suddenly die in a way that resembles intrinsic random death. Quiescent cells could also die. Dead cells were considered immediately as empty space and their position could be occupied by newborn or moving cancer cells. Cells might also randomly move in a neighboring site with a speed that was defined by the diffusion coefficient. Furthermore, cells differed phenotypically based on their cell-to-cell adhesiveness with more motile cells to prefer moving towards low dense areas and adhesive cells to prefer being near other cells, a hypothesis that has been proposed before to describe the invasion pattern of U87MG spheroids [23].

#### Parametrization of the Model

The initial tumor size was mapped based on the initial spheroid size according to the in vitro estimations. The initial cell density resembled the compactness of the tumor at the first time point and estimated the cellularity of the initial tumor. This parameter could not be derived from the experiments and was assumed to be equal to 90%. Thus, cells were initially seeded in a circular, relatively compact configuration where 90% of cells occupied the initial tumor area. The proliferation time of the cells was approximated by the relevant in vitro doubling time experiments. In order to proliferate, each cell must find an empty space for its daughter cell within the Moore neighborhood. If no vacancy was found in that area, the cell entered a quiescent state and kept searching until an empty space was found where it could immediately proliferate. In order to avoid possible synchronization artifacts in division, we randomly assigned an age to each cancer cell at the beginning of the simulations. Contact inhibition through space competition was simulated by controlling the proliferative depth of the spheroid, which resembles the maximum distance (measured in cells) over which a cell is able to push other cells away in order to divide [31]. To counterbalance proliferation and death among different conditions (PML and PML OE), it was assumed that random cell death was a given percentage of the proliferation rate. Drug mediated cell death was examined as phenomenon independent of proliferation rate. Once a cell died, the space became empty immediately and new cells could take its place.

Cell movement was stochastic and could adopt different motility mechanisms triggered by different cell-to-cell adhesion forces. Cell motility was considered as a resultant of the cell’s random diffusive movement capability, which was the same for the whole population, and its adhesive properties, which differed among phenotypes. To describe the diffusive movement, the diffusion Equation (1), adopted by [24] was discretized to movement probabilities for each individual cell. *Dc* and *C* in Equation (1) denote the diffusion coefficient and the cancer cells concentration, respectively. Cells were allowed to move in empty neighboring locations in the Moore neighborhood biased by their adhesive trait and according to the movement probabilities.

(2) $$\frac{\partial c}{\partial t}=\overset{\mathrm{diffusion}}{\overbrace{D_{c}\nabla^{2}c}}$$

Cells selected their preferred neighborhood according to their adhesive preference, which in general could vary between 0 (non-populated area) and 7 (highly populated area); a number depicting how many cells actually occupied the Moore neighborhood at which a cell moved. Motile cells were cells with low cell-to-cell adhesive preference and thus preferred empty neighborhoods, whereas adhesive cells had high adhesive preference, meaning that they were attracted towards highly populated areas [23]. In our work, *Motile* cells had preferences equal to 0 or 1 and *Adhesive* cells had preferences equal to 6 or 7. The adhesive property allowed a cell to move, only if its destination position fitted with its adhesive preference (Supplementary Figures S3 and S4). An arbitrary initialization on the spatial configuration of cellular phenotypes (adhesive and motile) was applied. Variation in our computational results was mainly derived from the randomness in the cellular movement and death process. Thus, all the simulations were repeated 10 times. In order to properly parametrize our mathematical model, a parameter study that investigates the extent at which each parameter affects tumor evolution was first conducted. The mathematical model was built and constrained in accordance with the experimental observations in a stepwise manner, starting from the growth condition and then continuing to the invasion experiments.

## 5. Conclusions

GB expansion is attributed to both excessive proliferation and local spreading. Due to its high infiltrative behavior, migratory GB sub-clones often evade current standard treatment, resulting in disease recurrence. New biomarkers reporting for the invasive capacity of the patient’s cancerous physiology could prove to be highly impactful in clinical practice, setting new targets in disease treatment. Our results are in line with previous findings, indicating these two major characteristics of GB evolution are modulated by distinct cellular mechanisms. Unravelling further the role of PML in GB progression could set PML as a therapeutic target, aiming at eliminating multiple sub-clones depending on their proliferative and/or invasive phenotype within the heterogeneous GB tumor.

Even though the employed algorithms describe a cancer predictive model that simulates tumor physiology and cellular phenomena [23,31], the model was further used to simulate molecular events in this work. The growth and invasive characteristics are intrinsic properties of the cells, not easily isolated in the in vitro experiments. Therefore, the mathematical model is utilized to discriminate and isolate the biological phenomena in order to study the specific effects of the PML protein in each condition. The biological experiments initialize the mathematical model and constraining the in silico tumor evolution to the initial in vitro conditions, the observed physiology is in line with the biological findings.

Along with the identification of specific biomarkers, an overall better understanding of GB evolution into the brain parenchyma could provide new potential therapeutic strategies along with existing ones to address GB’s infiltrative nature. To tackle questions related to cancer heterogeneity and treatment, mathematical and computational models accounting for specific physiological parameters can assist in this process. Such in silico models built in a minimalistic, stepwise manner and tightly constrained by the biological observations could provide important knowledge regarding the underlying mechanisms of study and a holistic overview of tumor progression. By taking advantage of the in silico insights into the in vitro procedures and the in vivo phenomenon, we could guide biological experimentation, better understand tumor biology and ultimately provide new platforms for personalized medicine and good quality cancer care of patients.

Under the light of precision medicine where variation in the expression levels of PML within a tumor and/or across different patients is expected, exploring the differential expression and effect of PML in patient-derived glioblastoma cell lines comprises an important next step. In this line, computational models capable of predicting tumor evolution and treatment, while accounting for phenotypic heterogeneity based on data derived from patient-derived GB cell lines, are both highly in demand and valuable [23,31,42].

## Figures and Tables

**Table 1:** Summary of the model fitted parameters under study for the non-invasive condition.

Parameter	U87MG	U87MG-PML OE
Proliferation Time	100 h	160 h
Cell Size	21.5 μm	21.5 μm
Initial Cell Density	90%	90%
Random Cell Death Rate	20% of the proliferation time	20% of the proliferation time
DZNeP-Mediated Cell Death Rate	0.0065 h^−1^	0.0065 h^−1^

**Table 2:** Summary of the model fitted parameters under study for the invasive condition, while the non-invasive parameters are kept constant.

Parameter	U87MG	U87MG-PML OE
Diffusion Coefficient	2.00 × 10^−9^ cm^2^/s	2.00 × 10^−9^ cm^2^/s
*Motile:Adhesive* Phenotypic Ratio	2:1	2:1
Diffusion Coefficient upon DZNeP Treatment	2.00 × 10^−9^ cm^2^/s	1.00 × 10^−9^ cm^2^/s
*Motile:Adhesive* Phenotypic Ratio upon DZNeP Treatment	1:1	1:4

## Data Availability

Data are available upon request.

## Supplementary Materials

The following are available online at https://www.mdpi.com/article/10.3390/ijms22126289/s1.
