# Supplementary material for: PML Differentially Regulates Growth and Invasion in Brain Cancer
Source: Int J Mol Sci. 2021 Jun 11;22(12):6289. doi: 10.3390/ijms22126289 (PMC8230868; doi:10.3390/ijms22126289)
Supplement: Supplementary file 1 [file ijms-22-06289-s001.zip › ijms-1243238-supplementary.pdf]

International Journal of
Molecular Sciences

MDPI

Article

PML Differentially Regulates Growth and Invasion in Brain Cancer

Maria Tampakaki [1,2,3], Mariam-Eleni Oraiopoulou [1], Eleftheria Tzamali [1], Giorgos Tzedakis [1], Takis Makatounakis [4], Giannis Zacharakis [3,*], Joseph Papamatheakis [4,5*] and Vangelis Sakkalis [1,*]

1 Institute of Computer Science, Foundation for Research and Technology-Hellas, GR-70013, Heraklion, Crete, Greece; mairata@ics.forth.gr (M.T.); marilena@ics.forth.gr (M.-E.O.); tzamali@ics.forth.gr (E.T.); gtzedaki@ics.forth.gr (G.T.)
2 School of Medicine, University of Crete, GR-71003, Heraklion, Crete, Greece
3 Institute of Electronic Structure and Laser, Foundation for Research and Technology-Hellas, GR-70013, Heraklion, Crete, Greece
4 Institute of Molecular Biology and Biotechnology, Foundation for Research and Technology-Hellas, GR-70013, Heraklion, Crete, Greece; makatou@imbb.forth.gr
5 Department of Biology, University of Crete, GR-70013, Heraklion, Crete, Greece;
* Correspondence: zahari@iesl.forth.gr (G.Z.); papamath@imbb.forth.gr (J.P.); sakkalis@ics.forth.gr (V.S.)

Supplementary Materials:

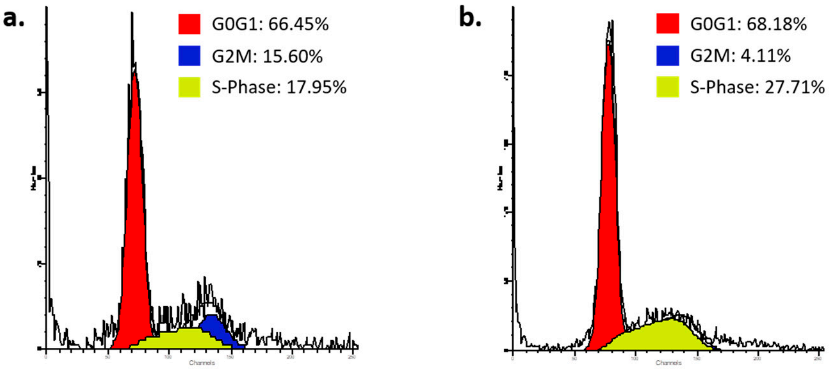

Supplementary Figure S1. Cell cycle distribution analysis by flow cytometry. Cell cycle analysis of (a) non-induced U87MG and (b) U87MG-PML OE cells.

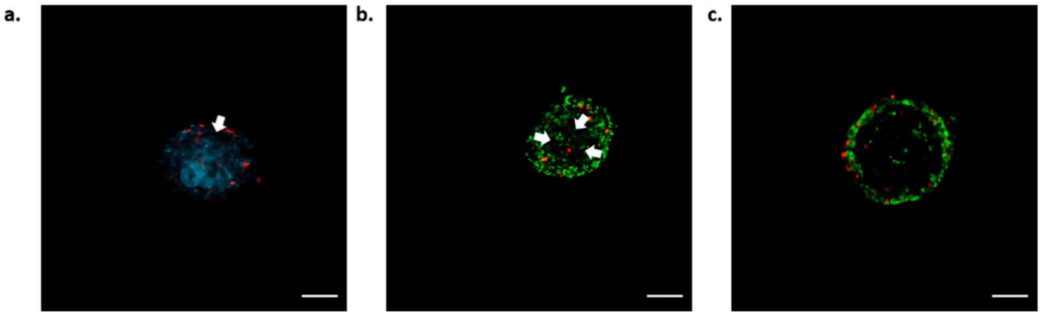

Supplementary Figure S2. Suppressed aggregation capacity under PML OE. (a) A central confocal slice one non-induced U87MG and (b,c) two U87MG-PML OE spheroids. White arrows represent vacant areas inside the spheroids. It is observed that these areas increase in size and number under PML OE. Cyan (H_2DCFDA) represents the cell bodies, red (DRAQ7) represents the dead nuclei and green (DsRed) represents the PML IV protein. Slices are at 40μm depth and scale bar is set at 100μm.

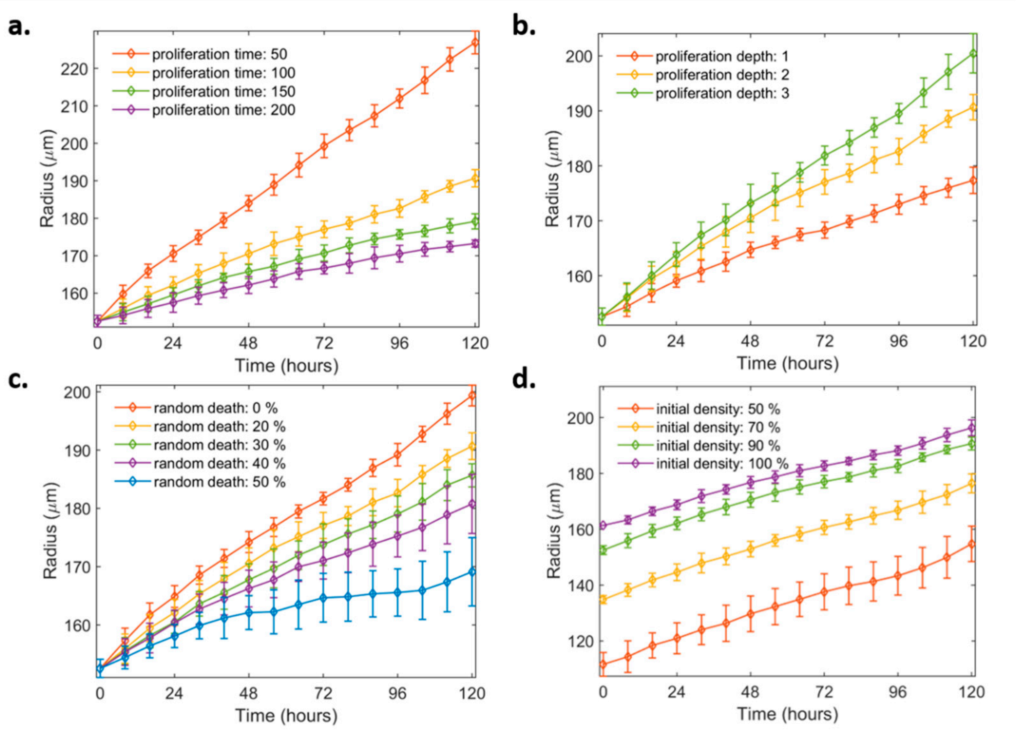

Supplementary Figure S3. Parameter study regarding the non-invasive condition. Each curve depicts the extent to which the different parameters affect tumor evolution in the invasion-inhibiting conditions. The respective *in vitro* radial expansion curves of the non-induced U87MG cells are used as reference. Parameter study regarding the role of (a.) proliferation time (b.) proliferation depth (c.) random cell death rate and (d.) initial cell density of the spheroid.

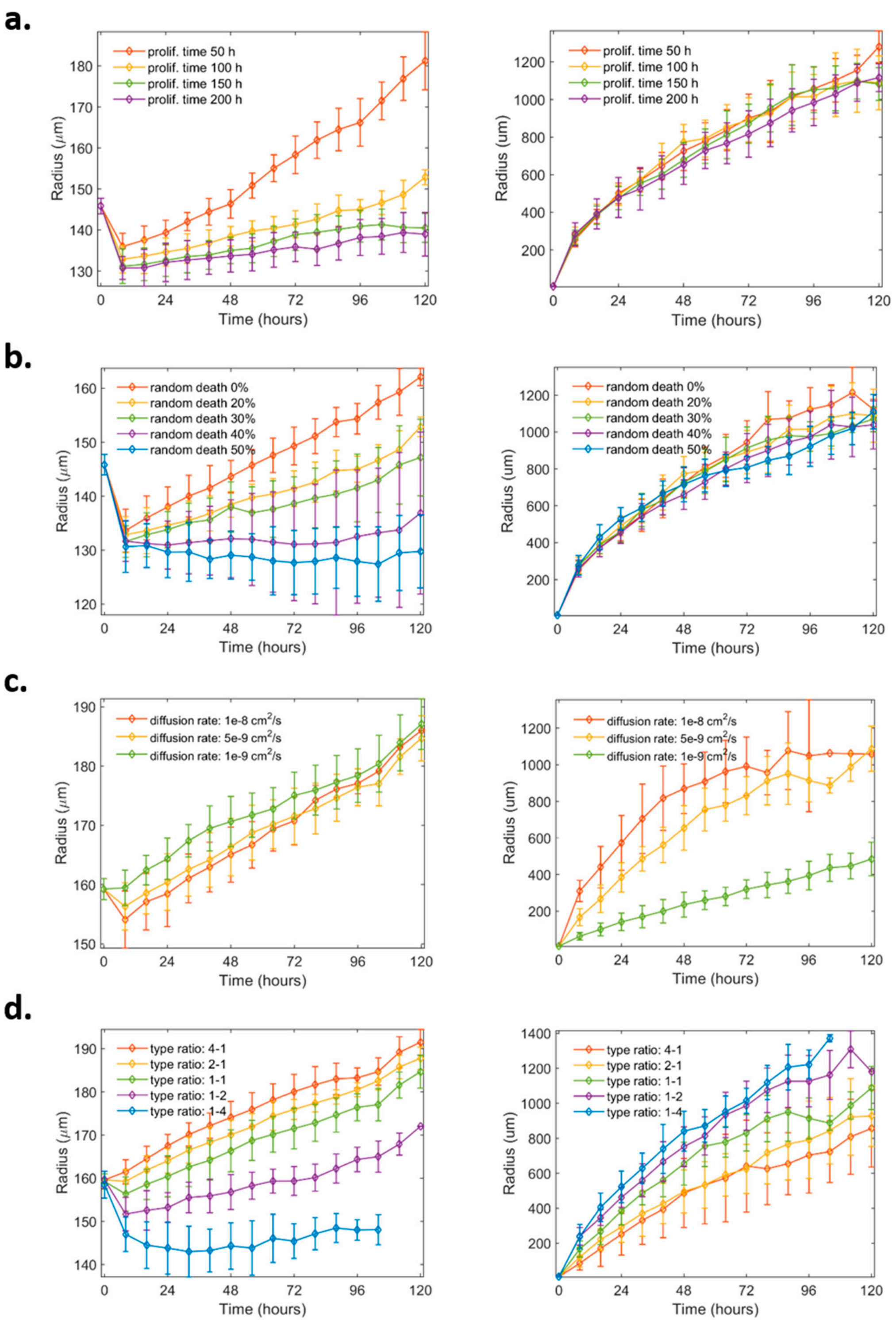

Supplementary Figure S4. Parameter study regarding the invasive condition. Each curve depicts the extent to which the different parameters affect the invasive dynamics of the tumor core and invasive rim of the spheroids. The respective *in vitro* radial expansion curves of the non-induced U87MG cells are used as reference. Parameter study regarding the role of (a.) proliferation time (b.) random cell death rate (c.) diffusion coefficient and (d.) seeding population phenotypic ratio regarding the adhesive and motile properties.

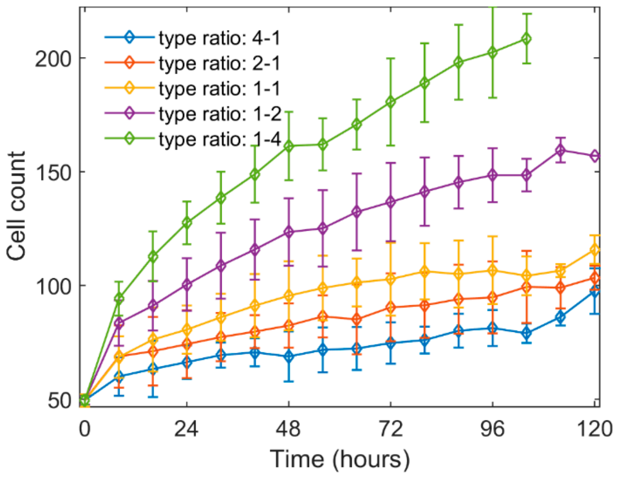

Supplementary Figure S5. *In silico* cell count of the density of invasive rim curves over time according to each phenotypic ratio simulation.
